# Different brain regions support deliberation during food choice in disordered and healthy eating

**DOI:** 10.21203/rs.3.rs-7753169/v1

**Published:** 2025-10-28

**Authors:** Alexandra F. Muratore, Eileen A. Hartnett, Karin Foerde, Blair W. Uniacke, B. Timothy Walsh, Joanna E Steinglass, Daphna Shohamy, Akram Bakkour

**Affiliations:** 1 New York State Psychiatric Institute and Department of Psychiatry, Columbia University Irving Medical Center, New York, NY 10032, USA.; 2 Department of Psychology and Mortimer B. Zuckerman Mind Brain Behavior Institute, Columbia University, New York, NY 10027, USA.; 3 Department of Psychology, University of Amsterdam, Amsterdam, the Netherlands.; 4 The Kavli Institute for Brain Science, Columbia University, New York, NY 10027, USA.; 5 Department of Psychology, Institute for Mind and Biology, and Neuroscience Institute, University of Chicago, Chicago, IL 60637, USA.

**Keywords:** anorexia nervosa, decision-making, food choice, restrictive eating, fMRI, computational psychiatry

## Abstract

The brain is wired to drive behavior towards foods that are high in sugar and fat. This natural pattern is reversed in individuals with anorexia nervosa (AN), who prefer low-sugar and low-fat foods to the point of starvation and even death. Here, we aimed to understand how changes in the brain contribute to the pattern of maladaptive food-related decisions in AN. We combined decision-making tasks with computational modeling of behavior and fMRI to examine food and non food-related decisions in individuals with AN and healthy controls (HC). Results from this pre-registered study suggest that patients with AN employ a decision process that relies on sampling and evaluating evidence that is similar to that in healthy individuals, regardless of the type of decision. However, when deliberating about what to eat, while HC engage the hippocampus, individuals with AN engage the striatum in addition to the hippocampus, apparently as sources of evidence in the decision process. These findings suggest that the maladaptive reversal of preferences in AN is related to reliance on different inputs to the process that leads to choice of food, rather than a maladaptive decision process *per se*.

People persistently make unhealthy choices, even when they know better. To better understand why people make maladaptive decisions, research over the past several decades has focused on the neurobiology of reward-seeking behavior. Our brains are wired to seek natural rewards, such as sweet and fatty foods [1–4]. Maladaptive decisions are often assumed to be the result of something going awry in an otherwise adaptive system, such as an overly active drive toward rewards or an imbalance between reward-seeking and behavioral inhibition [5–9]. This framework has helped understand some cases of maladaptive behavior. However, it has left open a fundamental gap in understanding why, in some cases, maladaptive behavior reflects the opposite pattern — a perplexing tendency to reject natural rewards, rather than to seek them. A complete understanding of motivated behavior must be able to explain the neural and cognitive mechanisms by which rejecting natural rewards can, in some cases, become rewarding.

Anorexia Nervosa (AN) is an eating disorder that presents such a pattern. In AN, the fundamental food preferences that are preserved across species are reversed. In contrast to the natural draw to foods that are high in sugar and fat, individuals with AN prefer low-calorie and low-fat foods, to the point of starvation and even death [10–12]. As with other persistent maladaptive behaviors, patients with AN are typically aware of the health risks, but are nonetheless unable to break the cycle [13–16]. A better understanding of how individuals with AN value food and make decisions about what to eat may therefore provide a new perspective on how the brain makes reward-based decisions and of how this process goes awry in disease.

Maladaptive decision-making may be driven by altered reliance on different sources of evidence (e.g., differences in inputs), different decision-making processes (e.g., differences in the way inputs are transformed into output), or both. Studies of food restriction in AN have begun to quantify both the behavioral and neural mechanisms underlying food choice [14–23]. The tendency to make maladaptive food choices in AN is assumed to be at least partly attributed to differences in valuation, which shift the inputs to the decision-making process. In other words, the preference of patients with AN for low-calorie, low-fat foods can lead to downstream consequences in how they deliberate between choice options [14,16]. This simple idea captures the basic clinical feature of AN and frames it in terms of normative models of decision-making. However, this observation also poses an experimental challenge. Focusing on the problem at the very initial stage of valuation can make it difficult to determine whether, and how, subsequent stages of decision-making may also be affected in AN. When accounting for the valuation differences, do patients with AN additionally differ in the *process* by which food-related decisions are made?

The goal of dissociating valuation from other aspects of decision-making becomes especially important when considering what is known so far about the neurobiology of decision-making in AN. Functional imaging studies have found increased striatal activity in individuals with AN when they are making decisions about food, relative to HC [18–20]. In one study of patients with AN [19], individuals completed a task in which they made a series of decisions about which food to eat. On each trial, they were asked to select between a neutrally-rated “reference” item and an alternative food item. Decisions about food correlated with BOLD activity in the dorsal striatum [19]. In addition, patients and controls differed in functional connectivity between the striatum and dorsolateral prefrontal cortex (dlPFC): patients showed increased striatal-prefrontal connectivity when choosing among low-fat items and reduced connectivity when choosing between high-fat items. Changes in dorsal striatal activation during food choice were also found to track with changes in treatment for AN: increases in the proportion of high-fat choices were related to a decrease in dorsal striatal activity during the food choice task [18,20].

Altogether, studies so far suggest that when individuals with AN make decisions, they display different patterns of behavior and show altered brain activity, especially in the striatum. Studies also found that these alterations relate to what patients with AN choose to eat, even outside of the experiment. The goal of the current study was to try to address the critical open question of what is driving these differences. How does the *process* by which decisions about what to eat are made contribute to maladaptive food choice in AN?

We were interested in disentangling two possible explanations. One is that patients with AN draw on different information to resolve their choice but subsequently follow a decision process that transforms information into action in a way that is similar to that of healthy individuals. On this view, individuals with AN and HC may have different *inputs* to the decision process (e.g., the subjective values placed on food options), but the process itself unfolds similarly across groups. Alternatively, it may be that in addition to differences in food values (i.e., the inputs), the decision-making process and its neural underpinnings may also differ between patients with AN and HC.

In studies of the mechanisms of decision-making, one powerful approach has been to focus on reaction time (RT) as a window into the decision process. Among healthy individuals, choice between two alternatives is well accounted for by a computational framework that posits that individuals sequentially sample and accumulate evidence (or information) in favor of and against choice options under consideration until a threshold, or boundary, is reached, at which point a decision is made [24–26]. In a decision of whether a moving car is green or blue, decision-making is difficult when the car is painted teal (i.e., there is roughly equal evidence for blue and green in the stimulus). In this case, a decision maker will take a long time to commit to a choice. Decision-making becomes easier and faster the closer the car’s color is to pure green (i.e., stronger evidence for green) or pure blue (i.e., stronger evidence for blue). Analogously, in a decision between two foods, choice of what to eat is difficult when the two foods are equally palatable (i.e., there is roughly equal evidence for the two foods). Choosing between a highly desirable food (i.e., one that holds a high subjective value) and a less desirable one (i.e., one that holds low subjective value), on the other hand is easy. In the latter case, there is no need for extensive deliberation — it is a no-brainer — and a decision can be made quickly. In other words, as the amount of evidence (i.e., the difference in subjective value between two choice options) increases, decision-making becomes “easier”, reflected in increased choice consistency and faster RTs [27,28]. A popular instantiation of this sequential sampling framework that captures the decision process (the drift diffusion model) has been used to explain both choices and RTs across a variety of forced-choice tasks, including those that require decisions about perceptual aspects of stimuli (e.g., “is the car green or blue?”) [29–32] and those that require a decision based on subjective value (e.g., “do I prefer the apple or the brownie?”) [27,33,34]. Thus, the drift diffusion model can be used to test whether the process by which patients with AN make decisions about food is the same as or differs from that of HC. If patients and healthy individuals follow a similar process to make decisions, we would expect that choices and decision time would conform to the regularities of the drift diffusion model. Should patients differ in the decision-making process, we would expect choices and deliberation time of patients with AN may not conform to regularities of the model (i.e., the model would fit the data poorly). The latter would suggest that maladaptive food choice in AN arises from differences in *both* input and process of decision-making about what to eat. Finally, if the decision process is different in individuals with AN from that in HC, it is important to better understand whether the difference is domain-general or specific to the food domain.

Food and non-food decision-making tasks can also be used to test whether neural processes underlying evidence accumulation differ between patients with AN and healthy individuals. Recent research examining the neural processes underlying decision-making in healthy individuals provides evidence for the role of the hippocampus in value-based decisions. Among healthy adults, BOLD activity within the hippocampus was more strongly correlated with decision time during value-based versus perceptual decisions [32]. These findings point to the role of the hippocampus in deliberative processes involving construction of value-based evidence. While other findings point to a role for the striatum in food choice among patients with AN, whether these individuals also rely on the hippocampus for evidence accumulation during value-based decisions remains unclear.

Here, we sought to separate out the valuation of food items from the process of decision-making. We compare food valuation and food-related decisions in individuals with AN and in HC. To determine whether any differences in the decision-making process are specific to food, we contrast the process of decision-making about food items with decisions about visual inputs. Finally, we use a sequential sampling framework and fMRI to determine whether patients with AN apply a different approach and use differential neural circuits when making decisions about food versus visual inputs.

Study design and hypotheses were pre-registered using the Open Science Framework (https://osf.io/jh3sd). We predicted that the groups would differ in how they make decisions about food but not in how they make decisions about visual inputs. We further predicted that fMRI would reveal that the groups differ in patterns of BOLD responses during food-related decision-making but not during perceptual decisions. Based on prior research [18–20,32], we expected that RTs would correlate positively with striatal activity among patients with AN, while hippocampal activity would correlate with RT among HC, which would reflect differences in how the groups draw on sources of evidence to resolve decisions about food. Better understanding of the differences that drive maladaptive food choice in a sample of patients with AN and HC may serve to further elucidate the cognitive neuroscience of decision-making underlying both normative and non-normative decisions.

## Results

**Figure 1:**
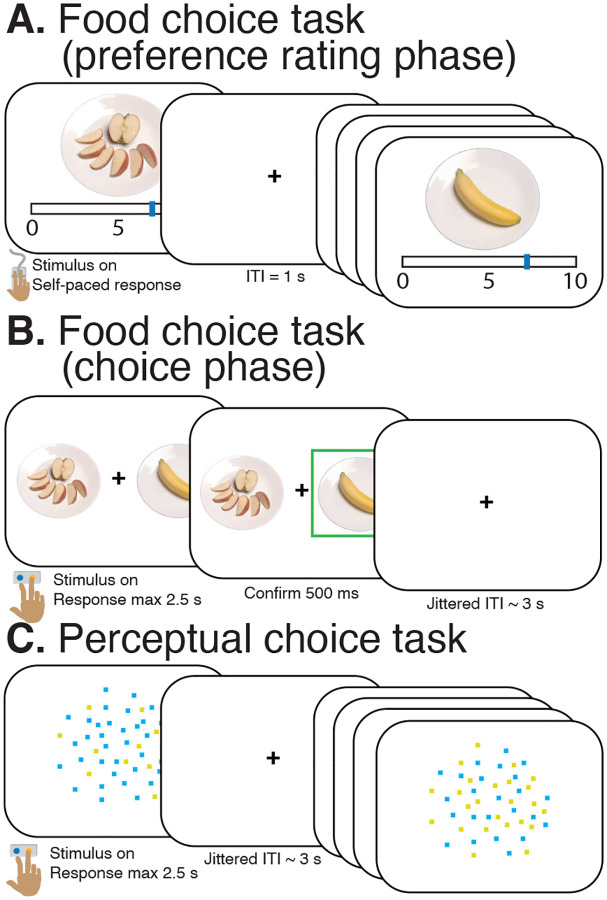
Task procedure. **A.** Participants provided preference ratings for 60 foods on a scale from 0 (least preferred) to 10 (most preferred). Nutritional information for each item is listed in the Food Folio dataset [48]. **B.** During the food choice task, participants were instructed to decide between a pair of items on each trial. **C.** During the perceptual choice task, participants were instructed to decide whether there were more blue dots or more yellow dots in a dynamic display of flickering dots.

We scanned HC (final *n* = 29) and patients with AN (final *n* = 30) while they performed two different decision-making tasks: (i) a preference-based task that involved choices between pairs of food items (“food choice task”; Figure 1A, 1B) and (ii) a control task that involved choices between two color options based on a visual display (“perceptual choice task”; Figure 1C). We examined differences in behavior (choice and RT), drift diffusion model fits, and BOLD activity between the tasks and between groups.

The study was pre-registered. For the ease of reading, we present the results below in narrative form, followed by a comprehensive summary of the pre-registered results and unexpected results in Table 4.

### Participants

Thirty-one patients with AN and thirty-one HC were recruited for participation in the study. This sample size was less than our pre-registered plan to test forty five participants per group because of the impact of the COVID-19 pandemic, which led a prolonged pause in data collection and significantly impaired our ability to recruit participants even after data collection resumed. Three participants were excluded from analysis due to excessive motion (*n* = 1 HC), insufficient data (*n* = 1 HC), and substance use during treatment (*n* = 1 patient with AN). The final sample consisted of 30 patients with AN and 29 HC (see Table 1 for participant demographics).

### Behavioral Analyses

#### Patients with AN choose different foods than HC, but do so using a similar decision-making process

**Figure 2.**
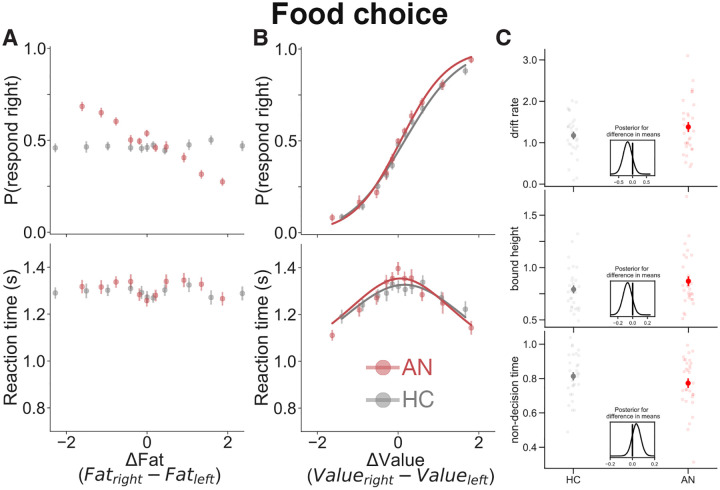
Individuals with AN make decisions that are more influenced by the fat content of the food, but still conform to regularities of the drift diffusion model, as do HC. Choices (top) and RT (bottom) as a function of (A) the difference in fat content between choice options and (B) the difference in subjective value ratings between choice options. (C) HC and patients with AN did not differ in key drift-diffusion model parameters fit to their choice and RT data. Dark points are means (error bars are standard error of the mean); light dots are individual participant data; colored solid lines are fits of the drift-diffusion model to the data. The posterior distributions of the Bayesian Estimation Supersedes the t-Test (BEST) tests (insets) indicate there are no credible differences in means between the two groups.

Patients with AN consistently chose low-fat foods and avoided high fat foods, a pattern that differed from HC, as expected (Figure 2A). This finding is consistent with the clinical profile and confirms the validity of the food-based decision task in capturing the hallmark maladaptive behavior of AN (see also [13,15,16,20,21]).

The food choice task design allowed us to account for different baseline preference ratings to probe whether and how the decision-making process unfolds in patients independent of the different choices. Overall, we found that the process by which decisions are made — indexed by choice accuracy, RT, and model fits — did not differ between the groups (Figure 2B & 2C).

Using each participant’s own subjective valuation, we found that both HC and patients with AN made choices that were consistent with their initial subjective valuation, as determined by the pre-task preference rating. As expected, patients generally rated foods as lower than controls, but both groups utilized the full rating scale (see Figure S1 for distribution of baseline preference ratings across groups). Next, we examined how the pattern of choices was affected by the difference in value between the two options on each trial (i.e., Δ*Value*; see Figure S2 for distribution of Δ*Value* across groups). We found that the consistency in behavioral choices increased as the difference in ratings between the items in a pair increased (main effect of Δ*Value* in Table 2, Figure 2B top). In other words, both groups were more consistent for ‘easier’ decisions, between options that were more different in value, than for ‘harder’ decisions, in which the two options were more similar to each other in value.

Another way to quantify the effect of choice difficulty is in RT. RTs for both groups were faster for ‘easier’ than ‘harder’ decisions (i.e., main effect of |Δ*Value*| on RT, Table 2, Figure 2B bottom), though this relationship was more strongly negative among patients with AN (interaction between |Δ*Value*| and Group on RT, Table 2). There were however no overall differences between patients with AN and HC on choices or RTs (no main effect of Group, Table 2). These findings were replicated in an independent sample of participants (Table S1).

The drift diffusion model was fit to choices and RTs from the food choice task for each participant and Δ*Value* was entered as a proxy for evidence strength for each trial. The model accounted for patterns of behavior in both groups of participants equally well (solid lines in Figure 2B). Furthermore, there were no differences in model parameters between groups (Figure 2C). These findings suggest that there were no differences between HC and patients with AN in *how* they engaged in a decision process about what to eat. In other words, even though the input to the decision process (i.e., the subjective values placed on food options) was different, the process by which that input is transformed into a behavioral output appears to be the same.

#### Patients with AN do not differ from HC in perceptual decision-making

The perceptual decision-making task offered an opportunity to assess behavior on a control task that shares many of the same features — trial-by-trial decisions, variation in choice difficulty — but for decisions that are based on visual features rather than food preferences.

**Figure 3.**
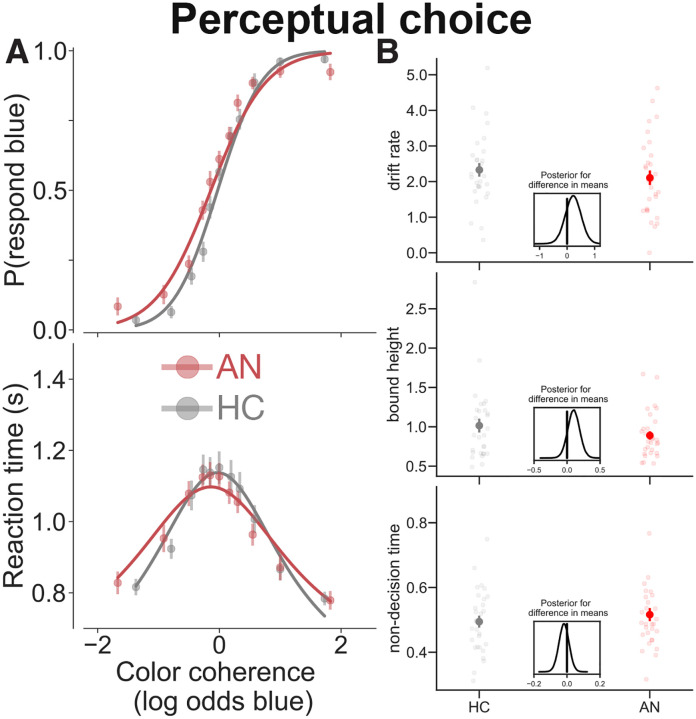
Patients with AN and HC do not differ in the way they make perceptual decisions. (A) Choices (top) and RTs (botton) are plotted against color coherence. Plotted on the top panel is the proportion of decisions where participants responded blue. Plotted on the bottom panel is RT. (B) HC and patients with AN did not differ in key drift-diffusion model parameters fit to their choice and RT data. Data for patients with AN are plotted in red, that of HC is plotted in gray. Points are means (error bars are standard error of the mean); light dots are individual participants; solid lines are fits of the drift-diffusion model to the data. The posterior distributions of the BEST tests (insets) indicate there are no credible differences in means between the two groups.

As expected, we found that individuals with AN made similar choices and had similar RTs to HC on the perceptual choice task. Both groups were more accurate when the display of dynamic dots stimulus contained many more yellow (highly positive values of color coherence) or many more blue dots (very negative values of color coherence; main effect of color coherence in Table 3 and Figure 3, top). Similarly, RTs for both groups were shorter for decisions on these ‘easier’ trials (Table 3 and Figure 3, bottom). There were no significant group differences on choice or RT and the effect of color coherence on choice and RT did not differ by group, though there was a marginal interaction between color coherence and group on choice (Table 3). Perceptual task choices and RTs were equally well described by the drift-diffusion model in HC and those with AN, and there were no group differences in the drift-diffusion model parameters between groups (Figure 3B). These findings were consistent with our *a priori* hypotheses, indicating that patients did not differ from healthy individuals in the behavioral process by which they made decisions based on perceptual features of the external environment. These findings were replicated in an independent sample of participants (Table S2). Moreover, the parallel results between groups confirm that the perceptual task offers a valuable control condition against which to compare brain-related activity in individuals with AN.

### fMRI Analyses

#### Similar representation of subjective value in the brain for both groups

**Figure 4.**
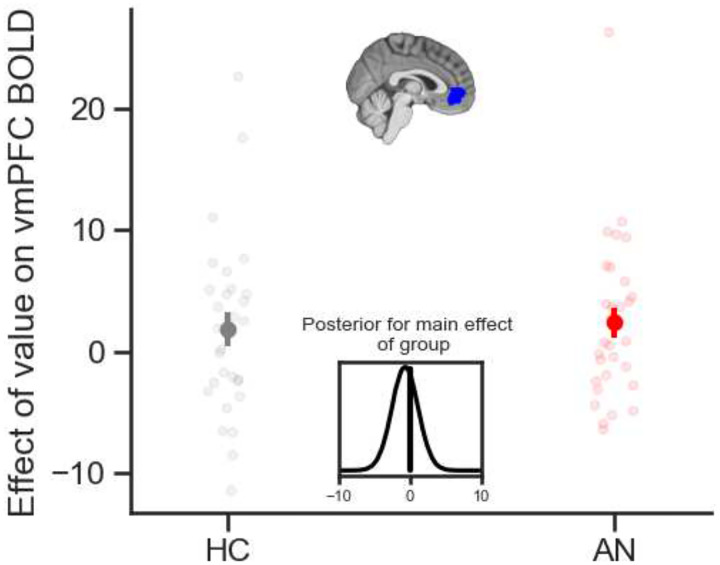
Value-related BOLD activity does not differ between individuals with AN and controls. The average value of the food items presented during each trial of the food choice task correlates with BOLD activity in the ventromedial prefrontal cortex (vmPFC) in patients with AN (red) and weakly in HC (gray). Run-level activitions were extracted from the vmPFC region of interest shown in blue (top inset). Points are means and error bars are standard error of the mean. Dots are individual participant data. The posterior distribution of the main effect of group in a mixed-effects Bayesian regression (lower inset) indicates there is no credible differences between groups.

We first sought to test whether the groups differ in how the brain responds to subjective value during food-based decision making. To determine whether there are any differences in brain activity between individuals with AN and controls in BOLD activity related to subjective value we ran an analysis with the average value of the food items within each trial as a regressor and extracted participant-level parameter estimates from a pre-defined mask of the ventromedial prefrontal cortex (vmPFC), a brain region known to code for value. As shown in Figure 4, we found that activity in the vmPFC weakly correlated with subjective value in both healthy individuals and patients with AN (i.e., main effect of mean value on vmPFC BOLD across groups, M = 2.39, SD = 1.30, 94% Highest Density Interval (HDI) [−0.05 4.83]), with no difference between groups (i.e., no credible effect of group, M = −0.72, SD = 1.86, 94% HDI [−4.23 2.79]). Whole-brain and small-volume correction analyses that investigate the effect of food value on BOLD provide some additional evidence for this conclusion (Tables S3 & S4). This suggests that, while the valuation of specific food items deviates starkly between the groups, once these differences are accounted for by the task, the effect of subjective value on brain activity does not differ between the groups.

#### Deliberation of food-related decisions elicit activity in different brain systems among patients with AN and healthy individuals

**Figure 5.**
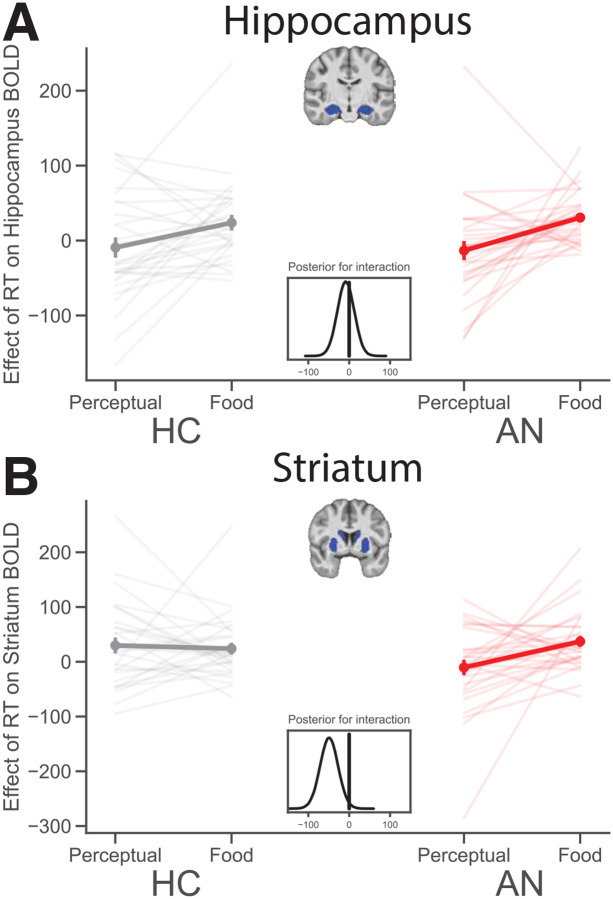
RT-related BOLD activity in the striatum differs between food- and perceptual decisions in AN when compared to HC. (A) RT correlated with BOLD activity in the hippocampus during food- but not perceptual decisions in both HC and AN. (B) RT correlated with BOLD acticity in the striatum during food- but not perceptual decisions in individuals with AN only. Points are means and error bars are standard error of the mean. Light-colored lines are individual participant data. The posterior distribution of the interaction between RT and task on BOLD in a mixed-effects Bayesian regression (lower insets) indicates a credible interaction only in the striatum.

We next examined choice-related activity in all participants, comparing decisions about food to decisions about visual input. We entered the time it takes to make decisions as a modulated regressor in our fMRI analysis to probe deliberation-related brain activation. We extracted parameter estimates for each task, run, and participant from *a priori* structural regions of interest to compare regional deliberation-related activation between types of decisions and groups in a Bayesian regression. Consistent with prior work [32], healthy individuals showed greater activation in the hippocampus during deliberation of food- when compared to perceptual decisions (Figure 5A left, M = 34.91, SD = 16.28, 94% HDI [4.23 65.39]). The same pattern is evident in the hippocampus for patients with AN (Figure 5A, right, M = 34.10, SD = 13.41, 94% HDI [17.73 68.28]) and there was no credible evidence of a difference in the hippocampus between healthy individuals and patients with AN (i.e., no interaction between task and group on deliberation-related hippocampus BOLD, M = −8.25, SD = 20.75, 94% HDI [−47.40 30.49]; see Figure 5A). Whole-brain and small-volume correction analyses partially support these ROI results (Tables S5 & S6). These findings bolster the idea that a relational memory system centered on the hippocampus supports deliberation of preference-based food choices, not only in healthy individuals, but also surprisingly in those with disordered eating.

In contrast, healthy individuals differed from patients with AN in their pattern of deliberation-related activation of the striatum (Figure 5B). Indeed, individuals with AN engaged the striatum during deliberation of food choices more than that of perceptual decisions (Figure 5B right, M = 44.89, SD = 15.17, 94% HDI [16.30 73.52]). Interestingly, and consistent with our predictions, the pattern of striatal activation in patients with AN differed from that in healthy individuals (i.e., credible interaction between task and group on deliberation-related striatum BOLD, M = −49.24, SD = 22.07, 94% HDI [−90.67 −7.56]). Whole brain analyses partially support the ROI findings (Figure S3, Tables S5 & S6). These findings suggest that, in addition to relying on the hippocampus as do healthy individuals, patients with AN also rely on the striatum during deliberation about what to eat. Thus, it appears that patients with AN rely on different brain systems during deliberation about what to eat when compared to healthy individuals.

#### Connectivity between striatum and dorsolateral prefrontal cortex (dlPFC) increases with food-choice decision time in patients with AN.

In addition to determining what brain regions were associated with timing of decisions for food-related versus perceptual-related decisions, we also investigated the functional connectivity between the striatum and other brain regions that vary with RT using a psychophysiological interaction (PPI) analysis. We used the PPI analysis to identify brain regions with activity that covaried with the activity of the striatum in a RT-dependent fashion. We hypothesized that, during food-related versus perceptual decisions, HC would show greater connectivity between the striatum and vmPFC, whereas patients with AN would show greater connectivity between the striatum and dorsolateral prefrontal cortex (dlPFC).

**Figure 6.**
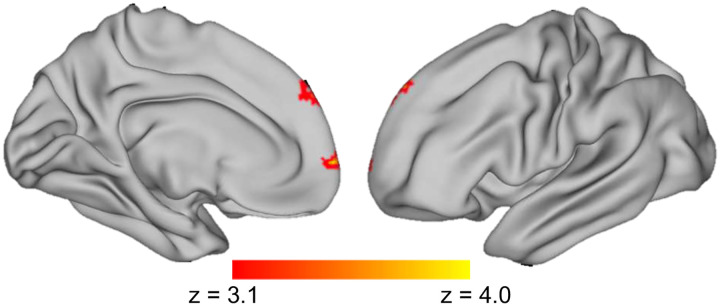
Patients with AN show greater RT-dependant dorsal fronto-striatal connectivity during value-based decisions. Medial (left) and lateral (right) view of a semi-inflated surface of the left hemisphere of a template brain, with results of the whole-brain PPI analysis among patients with AN projected onto cortical surface. Compared to the perceptual task data, patients’ RT-related activity during the food choice task was more strongly correlated with connectivity between the striatum and the middle frontal gyrus.

The results of the whole-brain PPI analysis are included in Figure 6 and Supplement Table S7. Contrary to our predictions, there was not a RT-dependent correlation between activity within the striatum and the vmPFC among HC. Note that there was no deliberation dependent activation of the striatum among HC (Figure 5B), which may help explain the lack of PPI results in HC. However, consistent with our predictions, whole-brain analyses revealed a significant RT-dependent correlation between the striatum and the middle frontal gyrus among patients with AN (Figure 6, Table S7). Although the middle frontal gyrus was selected as our pre-registered region of interest for the dlPFC, this region of interest included the midline and the significant cluster identified in the whole-brain analysis appears more medial than lateral. These results demonstrate that for patients, longer time to make decisions about food is associated with increased coupling between the striatum and the middle frontal gyrus. There were no three-way interactions between group, task and RT on striatal-dlPFC co-fluctuations, suggesting that there was no significant group difference in RT-related functional connectivity during food-related versus perceptual decisions.

Together, results of behavioral and imaging analyses indicate that patients make different choices about food than do HC, but there are more similarities than differences in the process of decision-making between the groups. After accounting for differences in subjective preferences, the patients show similar rational use of their own subjective values and their behavior conforms to a drift diffusion decision-making mechanism. However, we find that patients engage more activity in the striatum (in addition to the hippocampus) during deliberation of decisions about food than controls do. Altogether, this suggests that individuals with AN may draw on different sources of internal evidence during choice deliberation of food-based decisions.

## Discussion

The current study combined measures of decision behavior (RT and choice) with computational models and fMRI data to gain insight into the cognitive and neural processes underlying decision-making in patients with AN and healthy individuals. Contrary to our expectation, we found that patients with AN do not exhibit differences in the process by which they make decisions in either non-food (perceptual) or food-related tasks as compared to healthy individuals. Given the striking differences in what patients with AN choose to eat, these findings shift the focus of inquiry towards the input to the decision process that leads individuals to choose one food over another — i.e., the valuation stage.

Despite the similarities between groups in the way the decision process unfolds in both food and non-food-related decisions, our results provide some evidence of differences in the neural mechanisms underlying food-related decisions between the groups. Compared to decision time on the perceptual task, results indicate that patients’ deliberation time on the food choice task was more related to BOLD activity in the hippocampus and the striatum. Among HC, deliberation-related BOLD activation increased weakly in the hippocampus and not at all in the striatum for food- as compared to perceptual decisions. These results provide us with some evidence that patients with AN differ from HC in terms of the brain regions they engage during deliberation of food choice when compared to perceptual decisions. Given prior research showing that the hippocampus contributes to deliberation during food choice in healthy individuals [32], which we replicate here, it is interesting that patients with AN also show RT-related activation of the hippocampus during food choice when compared to perceptual decisions. Thus, in addition to the hippocampus, patients also show greater RT-related activation in the striatum, an effect that is credibly different than in HC. We interpret these fMRI results to suggest that patients with AN draw on different sources of internal evidence as input to a decision process, which leads them to value foods differently and make different choices about food when compared to healthy individuals [17–19,21].

To further explore this possibility, we set out to elucidate the brain regions that communicate with these brain regions implicated in deliberation during decisions about what to eat. Consistent with our pre-registered predictions, functional connectivity analyses show that BOLD time courses are more coupled between the striatum and dorsolateral prefrontal cortex during longer deliberations of food choice when compared to perceptual choice in patients with AN, but not in HC. Although connectivity analyses of fMRI data do not allow us to infer the direction of information flow between functionally coupled brain regions, we speculate that patients with AN may engage more control over which source of internal evidence to sample from during deliberation of food choice as the difficulty of the choice increases (i.e., the subjective value of the choice options are closer together, and RT is longer). This interpretation is consistent with the established role of dorsolateral prefrontal cortex in cognitive control processes [35,36].

Results of the current analyses extend previous research investigating neural mechanisms of food choice in AN by providing evidence to support the idea that maladaptive food choice among patients with AN is driven by differences in inputs to the decision process. An additional novel finding from this study is that, contrary to expectations, maladaptive food choice is *not* due to irregularities in the cognitive decision-making process itself. Findings of striatum and hippocampus engagement during deliberation of food relative to perceptual decisions contribute to a growing body of evidence suggesting that dorsal fronto-striatal circuitry underlies maladaptive food choice in AN [18–20], and further implicate both the striatum and hippocampus as contributing to decision-making among patients.

There are several limitations to the current study that are worth noting. The sample size of 29 HC and 30 patients with AN, while sizable, is smaller than the pre-registered sample and is thus likely underpowered to detect hypothesized whole-brain group differences. Moreover, as both the striatum and hippocampus are known to contribute to many different processes [37–39], it remains unclear what specific striatal or hippocampal-based processes are relied upon to guide decision-making differentially between groups. An additional limitation is the use of binary choice tasks; clinically-relevant decisions tend to be multi-alternative (e.g., deciding what snack to eat from a cabinet with multiple options), and thus, the generalizability of these findings may be limited. Additionally, the current study was conducted among a sample of patients voluntarily receiving highly specialized behavioral treatment for AN; as many patients with AN are not enrolled or engaged in intensive treatment settings, these results may not be representative of all individuals with AN. Future studies may wish to investigate the use of drift diffusion models or other computational models to explain multi-alternative choice decision-making among individuals with AN.

The current study is the first of its kind to combine tools from computational modeling of behavior and cognitive neuroscience tools to investigate the cognitive and neural mechanisms that underlie both perceptual and food choice decision-making among patients with AN. Results of this investigation provide initial evidence to suggest that patients follow a behavioral process of evidence accumulation during decision-making that is similar to healthy individuals, and extend evidence suggesting these individuals may differ in their recruitment of neural regions during the decision-making process, with increased engagement of the striatum during deliberation of food choice. A critical next step will be to further examine the neural and cognitive processes underlying the valuation stage of decision-making to determine whether, when, and how, valuation may change over the course of illness. Whether associations between food choice and striatal activity is a cause or result of the illness also remains unclear, and should be the focus of future investigation. Continued investigation of the mechanisms underlying food choice at each stage of the decision-making process may serve to elucidate the substrates of decision-making in both healthy individuals and psychiatric populations, and will potentially allow identification of targets for mechanism-based treatments.

## Online Methods

### Participants

All participants were right-handed females, ages 16 to 40 years, who had normal or corrected-to-normal vision, and were competent to provide informed consent or assent. Participants were excluded if they had a history of learning disability, concussion, seizure disorder, or other neurological disorder; were pregnant; were taking medication known to have acute effects on appetite (e.g., stimulants); or had contraindications to MRI. Patients with AN were receiving inpatient treatment at the New York State Psychiatric Institute and were eligible to participate if they had a DSM-5 [40] diagnosis of AN or atypical AN confirmed by the Eating Disorders Assessment (EDA-5) [41], were at a body mass index (BMI) of at least 14.5 kg/m^2^, and were medically stable. Comorbid diagnoses were assessed via Structured Clinical Interview for DSM-5 (SCID) [42]. Patients were excluded if they were experiencing acute suicidality or had a comorbid psychiatric diagnosis requiring specialized treatment. Due to high prevalence of mood and anxiety disorders among inpatients with AN, these comorbidities were not exclusionary. Healthy controls (HC) were recruited through flyers posted around Columbia University’s campuses and surrounding areas in New York City. HC were eligible to participate if they had a BMI between 18.5 and 25 kg/m^2^, had no current or past psychiatric diagnoses, as assessed by the EDA-5 and the SCID, and were not on psychotropic medications. Participant demographic information and clinical characteristics can be found in Table 1. The study was approved by the New York State Psychiatric Institute (NYSPI) and Columbia University Institutional Review Boards. Adults provided written informed consent and adolescents provided assent with parental consent prior to participation.

### Procedures

Patients with AN took part in the study on average 2.52 ± 2.14 weeks after hospital admission. All participants completed a standardized breakfast before completing a food choice task and a perceptual choice task during MRI scanning. The procedures are described briefly below and have been detailed extensively in a previous publication [32]. In addition, eyetracking data were collected during all tasks. Estimated IQ was assessed using the Wechsler Abbreviated Scale of Intelligence, second edition (WASI-II) [43]. Participants also completed psychological assessments that included the Eating Disorder Examination Questionnaire (EDE-Q) [44] the Three Factor Eating Questionnaire (TFEQ) [45], the State-Trait Anxiety Inventory (STAI) [46] and the Beck Depression Inventory (BDI) [47].

#### Experimental Tasks

##### *Food choice task* (Figure 1A–B):

The food choice task consisted of a series of food items presented during a preference rating phase and choice phase. Stimuli were taken from the Food Folio by Columbia Center for Eating Disorders stimulus set, a publicly available database of high-resolution color photographs of food items with corresponding nutritional content [48]. The preference rating phase was conducted outside the scanner prior to the start of the scan. In this phase, participants utilized a visual analogue scale to rate their preferences for 60 different food items from 0 (least prefer to eat) to 10 (most prefer to eat). These ratings were z-scored across ratings for each participant, which enabled unique pairing of foods, such that the difference in z-scored preference rating between each food item within a pair (*ΔValue = Rating*_*Item_on_right_z*_ - *Rating*_*Item_on_left_z*_) varied from pair-to-pair. In the choice phase, participants were presented with a series of decision trials, in which the pre-determined pairs of food items were presented in random order, one pair at a time, with each food item on one side of a fixation cross. On each trial, participants were instructed to select which item they preferred by pressing one of two buttons on an MRI-compatible button-box. Participants were given up to 3 s to make a response. Once a response was made, the selected food item was highlighted for 500 ms. Participant responses were considered “correct” when they selected the food that had previously received a higher rating during the preference rating phase, and “incorrect” when they selected the food that had previously received a lower preference rating. If no selection was made within the allotted time, the trial would end, and the message “Please respond faster” would appear on the screen for 500 ms. Inter-trial intervals (ITIs) were generated using the same procedure as the perceptual choice task. Participants were presented with a total of 210 trials across 3 runs (70 trials each, each 7 min). Prior to the task, participants were told that they would receive a snack consisting of their chosen food items from a single, randomly selected trial, to increase the likelihood that their choices would reflect true preferences. Patients were informed that, like all meals and snacks provided during treatment, the snack counted toward patient privileges. The snack item was provided after the scan.

##### *Perceptual choice task* (Figure 1C):

During the perceptual choice task, participants viewed a dynamic random dot display consisting of yellow and blue dots. Dots were presented at random locations within a central circular aperture (5 cm diameter). The proportion of blue to yellow dots was governed by color coherence, defined as the log odds that a dot is blue. Color coherence varied from trial-to-trial. Positive values of color coherence governed that more blue than yellow dots were present in the dynamic stimulus, while negative values of color coherence governed that more yellow than blue dots were in the stimulus. Additional details of this task can be found in a previous publication [32]. Participants were instructed to indicate, as quickly and accurately as possible, whether there were more yellow or more blue dots in the display by selecting one of two buttons on an MRI-compatible button-box. They were given up to 2.5 s to make a response. Once a response was made, the dots display disappeared and a central fixation cross reappeared. Trials were separated by a jittered ITI ranging from 1 to 12 s and averaged 3 s. Participants were first trained on the task with feedback until they reached an accuracy criterion of 80% or higher over the last four blocks of 10 trials. In the scanner, participants were presented with a total of 210 trials across 3 runs (70 trials each, each 6.5 min) and were not provided any feedback.

#### fMRI acquisition:

Imaging data were acquired on a 3T Siemens PRISMA MRI scanner with a 64-channel head coil. High-resolution structural images were acquired using a T1-weighted magnetization-prepared rapid acquisition gradient-echo (MPRAGE) three-dimensional sequence (repetition time (TR) = 2.3 s, echo time (TE) = 2.2 ms, flip angle (FA) = 8°, field of view (FoV) = 192 mm, matrix = 96 × 96). Functional data were acquired using a T2*-weighted echo planar imaging sequence (slice thickness = 2 mm, TR = 1.5 s, TE = 30 ms, FA = 68°, FoV = 192 mm, matrix = 96 × 96). Oblique axial slices aligned with the anterior commissure-posterior commissure line were acquired in an interleaved fashion. Each of the food choice runs consisted of 284 volumes, and each of the dots choice runs consisted of 264 volumes.

### Behavioral Analyses

#### Choice and RT:

Choices and RTs for both tasks were analyzed using repeated measures logistic and linear mixed effects regression models, respectively. In all models, participant and group were included as the random effects with a random intercept. For the dots choice task, choices were coded as 1 if participants chose blue or 0 if participants chose yellow. For the food choice task, choices were coded as 1 if participants chose the food item on the right side of the screen, or 0 if participants chose the item on the left side of the screen. Binary dots choice data were entered into a repeated measures logistic regression model to calculate the odds of selecting blue in the dots choice task based on task difficulty (*color coherence*) and group (HC vs. AN). RT data were entered into a linear regression model to determine the relationship between RT, dots choice task difficulty (|*color coherence*|), and group. Binary food choice data were entered into a repeated measures logistic regression model to calculate the odds of selecting the item on the right side of the screen in the food choice task based on difficulty (Δ*Value*), average value of food items, and group (HC vs. AN). RT data were entered into a linear regression model to determine the relationship between RT, food choice task difficulty (|Δ*Value*|), average value of food items, and group. Of note, we included average value of food items as a regressor to account for any differences in mean rating across items in a pair, though inclusion of this regressor in both models was a deviation from the pre-registration.

#### Drift-diffusion model:

To determine whether there are differences in the way participants in the two groups utilize evidence in service of decision-making, we fit choice and RT data simultaneously using a drift-diffusion model (DDM [24–26,49–51]). Briefly, the DDM assumes that when making a decision, participants sample evidence (from the external environment in the case of the dots task, from internal sources in the case of the food choice task) and integrate these samples of evidence over time until they reach one of two decision thresholds (blue or yellow for the dots choice task, right or left item for the food choice task). Once a threshold (or bound) is reached, the participant commits to a decision. Our model extended the basic DDM and allowed the decision threshold to vary over time (i.e., the bounds remained flat for a period then decreased over time, following an exponential function), allowed for a bias in the rate of evidence accumulation for one response versus the other, and allowed for the possibility of a non-linear monotonic relationship between the drift rate and stimulus strength (color coherence for the dots task and ΔValue for the food choice task). The details of the model and fitting procedure used here are detailed in a prior publication [32]. The model had eight free parameters: 1) the drift rate (*v*) governed the rate of evidence accumulation, 2) the initial bound height at the start of the decision (*B*_*0*_), 3) the delay before the bound starts to decrease (*B*_*del*_), 4) the coefficient of the exponential function that governs the shape of the decreasing bound (*B*_*2*_), 5) non-decision time related to perceptual and motor processing not related to the decision process itself is assumed to be a Normal distribution with a mean *t*_*nd*_, 6) and its standard deviation σ_tnd_, 7) a bias to the drift rate to correct the potential that the distribution of choices and RTs for left button presses is different than that for right button presses, 8) a coefficient for a power law function applied to stimulus strength that allows for a non-linear relationship between *v* and stimulus strength. These free parameters were first fit to data for all participants in each group and task separately. We also fit these parameters for each participant’s data for each task separately. The model was fit using maximum likelihood estimation. This model was previously validated and the parameters were found to be recoverable [32]. Individual participant parameter estimates were compared across groups using the Bayesian Estimation Supersedes the t Test (BEST) method [52]. BEST provided a posterior distribution for group mean comparisons for each of the three main parameters of interest: *v*, *B*_*0*_, and *t*_*nd*_. We interpreted an effect as credible if the 94% highest density interval (HDI) did not include 0.

### Imaging Analyses

#### Preprocessing

No preprocessing pipeline was specified in the pre-registration. Results included in this manuscript come from preprocessing performed using *fMRIPrep* 20.2.6 [53,54]; RRID:SCR_016216, which is based on *Nipype* 1.7.0 [55,56]; RRID:SCR_002502. Preprocessed data were smoothed using a 4 mm smoothing kernel full width at half maximum.

##### Anatomical data preprocessing

One T1-weighted (T1w) image was corrected for intensity non-uniformity (INU) with N4BiasFieldCorrection [57], distributed with ANTs 2.3.3 (Avants et al. 2008, RRID:SCR_004757). The T1w-reference was then skull-stripped with a *Nipype* implementation of the antsBrainExtraction.sh workflow (from ANTs), using OASIS30ANTs as target template. Brain tissue segmentation of cerebrospinal fluid (CSF), white-matter (WM) and gray-matter (GM) was performed on the brain-extracted T1w using fast (FSL 5.0.9, RRID:SCR_002823 [58]). A T1w reference map was computed after registration of 2 T1w images (after INU-correction) using mri_robust_template (FreeSurfer 6.0.1 [59]). Brain surfaces were reconstructed using recon-all (FreeSurfer 6.0.1, RRID:SCR_001847 [60]), and the brain mask estimated previously was refined with a custom variation of the method to reconcile ANTs-derived and FreeSurfer-derived segmentations of the cortical gray-matter of Mindboggle (RRID:SCR_002438 [61]). Volume-based spatial normalization to one standard space (MNI152NLin2009cAsym) was performed through nonlinear registration with antsRegistration (ANTs 2.3.3), using brain-extracted versions of both T1w reference and the T1w template. The following template was selected for spatial normalization: *ICBM 152 Nonlinear Asymmetrical template version 2009c* [62] (RRID:SCR_008796; TemplateFlow ID: MNI152NLin2009cAsym).

##### Functional data preprocessing

For each of the 6 BOLD runs per participant (across all tasks), the following preprocessing was performed. First, a reference volume and its skull-stripped version were generated using a custom methodology of *fMRIPrep*. A B0-nonuniformity map (or *fieldmap*) was estimated based on two (or more) echo-planar imaging (EPI) references with opposing phase-encoding directions, with 3dQwarp [63] (AFNI 20160207). Based on the estimated susceptibility distortion, a corrected EPI (echo-planar imaging) reference was calculated for a more accurate co-registration with the anatomical reference. The BOLD reference was then co-registered to the T1w reference using bbregister (FreeSurfer) which implements boundary-based registration [64]. Co-registration was configured with six degrees of freedom. Head-motion parameters with respect to the BOLD reference (transformation matrices, and six corresponding rotation and translation parameters) are estimated before any spatiotemporal filtering using mcflirt (FSL 5.0.9 [65]). BOLD runs were slice-time corrected to 0.708s (0.5 of slice acquisition range 0s-1.42s) using 3dTshift from AFNI 20160207 [63], RRID:SCR_005927. The BOLD time-series (including slice-timing correction when applied) were resampled onto their original, native space by applying a single, composite transform to correct for head-motion and susceptibility distortions. These resampled BOLD time-series will be referred to as *preprocessed BOLD in original space*, or just *preprocessed BOLD*. The BOLD time-series were resampled into standard space, generating a *preprocessed BOLD run in MNI152NLin2009cAsym space*. First, a reference volume and its skull-stripped version were generated using a custom methodology of *fMRIPrep*.

Several confounding time-series were calculated based on the *preprocessed BOLD*: framewise displacement (FD), DVARS and three region-wise global signals. FD was computed using two formulations following Power (absolute sum of relative motions [66]) and Jenkinson (relative root mean square displacement between affines [65]). FD and DVARS are calculated for each functional run, both using their implementations in *Nipype* (following the definitions by Power et al. [66]). The three global signals are extracted within the CSF, the WM, and the whole-brain masks. Additionally, a set of physiological regressors were extracted to allow for component-based noise correction (*CompCor* [67]). Principal components are estimated after high-pass filtering the *preprocessed BOLD* time-series (using a discrete cosine filter with 128s cut-off) for the two *CompCor* variants: temporal (tCompCor) and anatomical (aCompCor). tCompCor components are then calculated from the top 2% variable voxels within the brain mask. For aCompCor, three probabilistic masks (CSF, WM and combined CSF+WM) are generated in anatomical space. The implementation differs from that of Behzadi et al. [67] in that instead of eroding the masks by pixels on BOLD space, the aCompCor masks are subtracted a mask of pixels that likely contain a volume fraction of GM. This mask is obtained by dilating a GM mask extracted from the FreeSurfer’s *aseg* segmentation, and it ensures components are not extracted from voxels containing a minimal fraction of GM. Finally, these masks are resampled into BOLD space and binarized by thresholding at 0.99 (as in the original implementation). Components are also calculated separately within the WM and CSF masks. For each CompCor decomposition, the *k* components with the largest singular values are retained, such that the retained components’ time series are sufficient to explain 50 percent of variance across the nuisance mask (CSF, WM, combined, or temporal). The remaining components are dropped from consideration. The head-motion estimates calculated in the correction step were also placed within the corresponding confounds file. The confound time series derived from head motion estimates and global signals were expanded with the inclusion of temporal derivatives and quadratic terms for each [68]. Frames that exceeded a threshold of 0.5 mm FD or 1.5 standardized DVARS were annotated as motion outliers. All resamplings can be performed with *a single interpolation step* by composing all the pertinent transformations (i.e. head-motion transform matrices, susceptibility distortion correction when available, and co-registrations to anatomical and output spaces). Gridded (volumetric) resamplings were performed using antsApplyTransforms (ANTs), configured with Lanczos interpolation to minimize the smoothing effects of other kernels [69]. Non-gridded (surface) resamplings were performed using mri_vol2surf (FreeSurfer). First, a reference volume and its skull-stripped version were generated using a custom methodology of *fMRIPrep*. A B0 nonuniformity map (or *fieldmap*) was estimated based on a phase-difference map calculated with a dual-echo GRE (gradient-recall echo) sequence, processed with a custom workflow of *SDCFlows* inspired by the epidewarp.fsl script and further improvements in HCP Pipelines [70]. The *fieldmap* was then co-registered to the target EPI (echo-planar imaging) reference run and converted to a displacements field map (amenable to registration tools such as ANTs) with FSL’s fugue and other *SDCflows* tools. Based on the estimated susceptibility distortion, a corrected EPI (echo-planar imaging) reference was calculated for a more accurate co-registration with the anatomical reference. We did not acquire fieldmaps on all participants. For those participants missing a fieldmap, no fieldmap correction was performed within *fMRIPrep*. All other pre-processing steps for these participants were the same as those indicated above.

Many internal operations of *fMRIPrep* use *Nilearn* 0.6.2 [71], RRID:SCR_001362, mostly within the functional processing workflow. For more details of the pipeline, see the section corresponding to workflows in *fMRIPrep*’s documentation.

#### Regions of Interest:

As stated in the pre-registration, analyses included four bilateral regions of interest (ROIs), including the striatum, the hippocampus, the dorsolateral prefrontal cortex dlPFC), and the ventromedial prefrontal cortex (vmPFC). The striatum, hippocampus, and dlPFC ROIs were selected from the corresponding regions in the Harvard-Oxford Subcortical Probabilistic Atlas thresholded at 25% (HOSPA [72]). The striatum ROI was created using a combination of left and right caudate and putamen, the hippocampus ROI was created using a combination of the left and right hippocampus, and the dlPFC ROI was created using a combination of the left and right middle frontal gyrus. The vmPFC ROI used was selected from a meta-analysis of fMRI experiments examining neural correlates of subjective value (see Bartra et al., 2014 [73], for methods on ROI construction). All ROIs were transformed to the fMRI template space (MNI152NLin2009cAsym) at 1 mm resolution (HOSPA transformed atlas was accessed via Templateflow.org).

#### GLM model set-up

##### Food choice task:

We conducted a generalized linear model (GLM) analysis on the food choice task fMRI data (“pre-registered food GLM”) followed a pre-registered model, and 9 regressors of interest: (i) onsets for all correct choice trials, modeled with a duration equal to the average RT across all valid choice trials and participants; (ii) same onsets and duration as (i) but modulated by |Δ*Value*| demeaned across these trials within each run for each participant; (iii) same onsets and duration as (i) but modulated by RT demeaned across these trials within each run for each participant; (iv-vi) similar to regressors (i-iii) but for incorrect trials; (vii) onsets for all valid trials and same duration as all other regressors, modulated by demeaned average rating across both food items in a pair; (viii) onsets for all valid trials and same duration as all other regressors, modulated by indicator for left/right response; ix) onset for missed trials, with duration equal to the duration of the stimulus presentation (3 s). The pre-registered food GLM included six head-motion parameters (x,y,z translation and rotation), framewise displacement (FD), and DVARS as confound regressors. Volumes with FD > 0.5 or standard DVARS > 1.5 were scrubbed and included as nuisance regressors. These cut-offs were used to remain consistent with fMRIPrep’s current definition of motion outliers, though it was notably a deviation from the pre-registration, which indicated excessive motion would be defined as runs with > 4 mm head motion or volumes with FD or raw DVARS > 0.9. As was pre-registered, runs with > 25% “to-be-scrubbed” volumes were excluded from analyses. Participants with > 2 excluded runs were excluded from analyses.

##### Perceptual choice task:

GLM analysis on the perceptual choice task fMRI data followed the pre-registered model, and included eight regressors of interest: (i) onsets for all correct choice trials, modeled with a duration equal to the average RT across all valid choice trials and participants; (ii) same onsets and duration as (i) but modulated by |color coherence| demeaned across these trials within each run for each participant; (iii) same onsets and duration as (i) but modulated by RT demeaned across these trials within each run for each participant; (iv-vi) similar to regressors (i-iii) but for incorrect trials; (vii) onsets for all valid trials and same duration as all other regressors, modulated by indicator for left/right response; ix) onset for missed trials, with duration equal to the duration of the stimulus presentation (3 s). The GLM included six head-motion parameters (x, y, z translation and rotation), framewise displacement (FD), and DVARS as confound regressors. Volumes with FD > 0.5 or standard DVARS > 1.5 were scrubbed and included as nuisance regressors, which was a deviation from the pre-registered definition of excessive motion, as described above. As was pre-registered, runs with > 25% of volumes deemed “to-be-scrubbed” were excluded from analyses. Participants with > 2 excluded runs were excluded from analyses.

#### GLM model estimation and correction for multiple comparisons

Food and perceptual GLMs were estimated using FMRIB’s Software Library’s (FSL) FMRI Expert Analysis Tool (FEAT) v. 6.0. For each task, first-level time-series analyses were performed for each run for each participant. The first-level contrast images were then combined across runs per participant using fixed effects. Group-level analyses were conducted using mixed effects via FMRIB’s Local Analysis of Mixed Effects (FLAME 1) tool.

Consistent with the pre-registration, whole-brain group-level maps were corrected to control for multiple comparisons using an uncorrected cluster-forming threshold of z = 3.1 and corrected extent threshold of p < 0.05.

#### Contrasts of interest

##### Effect of Mean Value of Food Pairs:

The first contrast of interest was the effect of differences in mean value rating across items in a pair between trial types during the food choice task. We conducted whole-brain analyses in both groups to identify regions in the brain that show an effect of mean value, i.e., a correlation between mean value across pairs and BOLD activity during all valid trials for both tasks (regressor (vii) for pre-registered food GLM). Results were compared between groups and tasks to assess the interaction between group, task, and mean value of food pairs on BOLD activity within the whole-brain and within the vmPFC.

##### Effect of RT:

We next conducted whole-brain analyses in both patients with AN and HC to identify regions in the brain that show an effect of RT (i.e., a correlation between RT and BOLD activity during all valid trials for both tasks [regressors (iii) + (vi) for pre-registered food and perceptual GLMs]). We then compared brain regions that show an effect of RT between groups and tasks to assess the interactions between group (patients vs. HC), task (perceptual vs. food), and RT on BOLD activity within the whole-brain and, as specified in the pre-registration, within the hippocampus and striatum.

##### Psychophysiological Interaction (PPI):

Finally, as pre-registered, we conducted a psychophysiological interaction (PPI) analysis to identify brain regions that covaried with activity of the striatum. The striatum ROI was used as the seed in the PPI analysis. The striatum seed was first deconvolved to obtain the neuronal time-course and then multiplied with the task regressor and reconvolved to create the PPI regressor. For the perceptual choice task, we included 10 regressors in our GLM: (i-viii) same regressors included in the pre-registered perceptual GLM, (ix) the raw time course extracted from the seed; (x) the PPI regressor with the same onsets as i. For the food choice task, we included 11 regressors in our GLM: (i-ix) same regressors included in the pre-registered food GLM, (x) the raw time course extracted from the seed; (xi) the PPI regressor with the same onsets as i. For each task, we conducted a PPI analysis across the whole-brain and, as pre-registered, within the dlPFC and vmPFC and compared regional effects between groups and tasks.

#### ROI analysis of GLM estimates

In addition to the whole-brain analyses laid out above, we extracted the parameter estimates from pre-defined regions of interest for each task, run, and participant map for the contrasts of interest described above. The extracted parameter estimates were then entered into a mixed-effects linear regression fit in a Bayesian framework using the python package Bambi [74]. For analysis of the effect of subjective value on vmPFC BOLD, the model included group (AN or HC) as a regressor of interest. For the analysis of the effect of RT on BOLD, the model included task (food or dots), group (AN or HC), and the interaction between task and group as regressors of interest. For all models, a random intercept and a random slope (when applicable) was entered as random effects per participant. Bambi provided a posterior distribution for all fixed effects. We interpreted an effect as credible if the 94% highest density interval (HDI) did not include 0.

## Supplementary Material

This is a list of supplementary files associated with this preprint. Click to download.

- MuratoreANDMSsupplement.pdf
- MuratoreANDMSsupplement.pdf

## Figures and Tables

**Table 1:** Participant clinical characteristics.

	HC (n = 29)	AN (n = 28) / Atypical AN (n = 2)			
	Mean ± SD	Mean ± SD	t	df	^p^
**Age (years)**	25.13 ± 4.85 (18–37)	24.50 ± 6.52 (16–41)	0.43	53.54	0.67
**BMI (kg/m**^**2**^)	21.75 ± 1.62 (19.1–24.5)	17.48 ± 1.73 (14.5–21)	9.70	55.74	<.001
**Subtype**					
Restricting / Binge-purge	-	n = 16 / 14	-	-	-
**Duration of Illness (years)**	-	7.37 ± 7.18 (0.25–23)	-	-	-
**EDE-Q, Global Score**	0.23 ± 0.31(0–1.1)	4.42 ± 1.07 (1.39–5.9)	−20.44	34.74	<.001
**TFEQ-R**	4.37 ± 3.43 (0–16)	18.62 ± 2.82 (12–21)	−16.90	50.46	<.001
**STAI-T**	29.92 ± 7.54 (20–51)	62.55 ± 9.66 (39–80)	−13.93	51.53	<.001
**BDI**	1.35 ± 2.11 (0–7)	31.53 ± 9.82 (8–47)	−16.20	30.58	<.001
**WASI-II FSIQ**	114.48 + 9.12 (101–140)	107.97 + 2.48 (79–135)	2.17	50.91	.04
**Race/Ethnicity**	n (%)	n (%)			
African American	3 (10%)	0 (0%)			
Asian	11 (38%)	2 (.07%)			
Caucasian	10 (35%)	24 (80%)			
Hispanic	4 (14%)	3 (.1%)			
Biracial	1 (3%)	1 (.03%)			

BMI = Body Mass Index at time of study, EDE-Q = Eating Disorder Examination Questionnaire (value missing for 4 HC), TFEQ-R = Three Factor Eating Questionnaire, Restraint Scale (value missing for 2 HC and 1 AN), STAI-T = Spielberger Anxiety Index, Trait version (value missing for 4 HC and 1 AN); BDI = Beck Depression Index (value missing for 1 AN); WASI-II FSIQ = Wechsler Abbreviated Scale of Intelligence, 2^nd^ edition, Full Scale IQ score. Values were compared between groups using Welch’s t-test.

**Table 2.** Regression results for choice and reaction time during food choice task

	Chose Right			RT		
	*Odds Ratio*	*CI*	*p*	*β*	*CI*	*p*
Intercept	0.94	0.86 – 1.02	0.12	1.38	1.33 – 1.43	**<0.001**
ΔValue	5.83	4.33 – 7.85	**<0.001**	−0.11	−0.13 - −0.08	**<0.001**
Group [HC]	0.91	0.81 – 1.03	0.128	−0.05	−0.12 – 0.03	0.215
Avg Value	0.97	0.91 – 1.03	0.346	−0.11	−0.12 - −0.09	**<0.001**
ΔValue × Group	0.88	0.58 – 1.32	0.524	0.04	0.00 – 0.07	**0.026**
ΔValue × Avg Value	1.17	0.93 – 1.46	0.178	0	−0.02 – 0.02	0.971
Group × Avg Value	1.03	0.95 – 1.12	0.483	0.04	0.02 – 0.07	**0.002**
ΔValue × Group × Avg Value	0.99	0.74 – 1.33	0.958	0	−0.03 – 0.03	0.833
N	59			59		
Observations	11847			11847		
Marginal R^2^	0.357			0.081		
Conditional R^2^	0.426			0.214		

Note: for RT regression, Δ*Value* is entered as absolute value of signed Δ*Value*. Avg Value is included to account for differences in overall value, but its effect on behavior is not discussed.

**Table 3.** Regression results for choice and reaction time during perceptual choice task

	Chose Blue			RT		
	*Odds Ratio*	*CI*	*p*	*β*	*CI*	*p*
Intercept	1.66	1.26 – 2.18	**<0.001**	1.12	1.04 – 1.20	**<0.001**
Color Coherence	24.77	12.65 – 48.48	**<0.001**	−0.17	−0.21 - −0.13	**<0.001**
Group [HC]	0.83	0.56 – 1.22	0.347	0.02	−0.09 – 0.13	0.744
Color Coherence × Group	2.36	0.90 – 6.20	0.08	−0.02	−0.07 – 0.04	0.586
N	59			59		
Observations	11956			11956		
Marginal R^2^	0.622			0.086		
Conditional R^2^	0.801			0.308		

Note: for RT regression, Color Coherence is entered as absolute value of signed Color Coherence

**Table 4.** Pre-registered hypotheses and corresponding results.

Pre-registered Hypothesis	Result
Behavior on the food choice task will differ between patients with AN and HC.	The results (Figure 2B, Table 2, & Table S1) do not support this hypothesis.
Behavior on the perceptual task will be the same for patients with AN and HC.	The results (Figure 3, Table 3, & Table S2) support this hypothesis.
Eyetracking will reveal differences in gaze patterns in patients with AN and HC.	This hypothesis was not tested due to eyetracking data quality issues.
RT will correlate more positively with BOLD activity in the striatum during decisions about food compared to perceptual decisions in patients with AN.	The ROI analysis results (Figure 5B) and whole-brain fMRI analysis (Figure S3) support this hypothesis. The same effect was also found in the hippocampus (Figures 5A, S3). The effect in the hippocampus did not survive whole brain correction (Table S5).
RT will correlate more positively with BOLD activity in the hippocampus during food-based compared to perceptual decisions in HC.	The ROI analysis results (Figure 5A) support this hypothesis, but the effect did not survive whole-brain correction for multiple comparisons (Table S5).
Functional connectivity between striatum and dlPFC will increase with RT during food choice compared to perceptual choice in patients with AN.	The results support this hypothesis (Figure 6 & Tables S7, S8).
Functional connectivity between striatum and vmPFC will increase with RT during food choice compared to perceptual choice in HC.	The results do not support this hypothesis (Table S7).

## Data Availability

The data supporting the results reported in this article are maintained by the Eating Disorders Research Unit of the New York State Psychiatric Institute and are available upon request from the corresponding authors. The study was pre-registered on the Open Science Framework (OSF) at https://osf.io/jh3sd.
